# Ambient Air Pollution Exposure and Outcomes in Patients Receiving Lung Transplant

**DOI:** 10.1001/jamanetworkopen.2024.37148

**Published:** 2024-10-17

**Authors:** Olawale Amubieya, Sam Weigt, Michael Y. Shino, Nicholas J. Jackson, John Belperio, Michael K. Ong, Keith Norris

**Affiliations:** 1 Division of Pulmonary, Critical Care and Sleep Medicine, Department of Medicine, David Geffen School of Medicine, University of California, Los Angeles; 2 Statistics Core, Division of General Internal Medicine and Health Services Research, David Geffen School of Medicine, University of California, Los Angeles; 3 Division of General Internal Medicine and Health Services Research, Department of Medicine, David Geffen School of Medicine, University of California, Los Angeles; 4 Department of Medicine, Veterans Affairs Greater Los Angeles Healthcare System, Los Angeles, California; 5 Department of Health Policy and Management, Fielding School of Public Health, University of California, Los Angeles

## Abstract

**Importance:**

Elevated ambient fine particulate matter (PM_2.5_) air pollution exposure has been associated with poor health outcomes across several domains, but its associated outcomes among lung transplant recipients are poorly understood.

**Objective:**

To investigate whether greater PM_2.5_ exposure at the zip code of residence is associated with a higher hazard for mortality and graft failure in patients with lung transplants.

**Design, Setting, and Participants:**

This retrospective cohort study used panel data provided by the United Network for Organ Sharing, which includes patients receiving transplants across all active US lung transplant programs. Adult patients who received lung transplants between May 2005 and December 2016 were included, with a last follow-up of September 10, 2020. Data were analyzed from September 2022 to May 2023.

**Exposure:**

Zip code–level annual PM_2.5_ exposure was constructed using previously published North American estimates.

**Main Outcomes and Measures:**

The primary outcome was time to death or lung allograft failure after lung transplant. A gamma shared frailty Cox proportional hazards model was used to produce unadjusted and adjusted hazard ratios (HRs) to estimate the association of zip code PM_2.5_ exposure at the time of transplant with graft failure or mortality.

**Results:**

Among 18 265 lung transplant recipients (mean [SD] age, 55.3 [13.2] years; 7328 female [40.2%]), the resident zip code’s annual PM_2.5_ exposure level was greater than or equal to the Environmental Protection Agency (EPA) standard of 12μg/m^3^ for 1790 patients (9.8%) and less than the standard for 16 475 patients (90.2%). In unadjusted analysis, median graft survival was 4.87 years (95% CI, 4.57-5.23 years) for recipients living in high PM_2.5_ areas and 5.84 years (95% CI, 5.71-5.96 years) for recipients in the low PM_2.5_ group. Having an annual PM_2.5_ exposure level greater than or equal to the EPA standard 12 μg/m^3^ was associated with an increase in the hazard of death or graft failure (HR, 1.11; 95% CI, 1.05-1.18; *P* < .001) in the unadjusted analysis and after adjusting for covariates (HR, 1.08; 95% CI, 1.01-1.15; *P* = .02). Each 1 μg/m^3^ increase in exposure was associated with an increase in the hazard of death or graft failure (adjusted HR, 1.01; 95% CI, 1.00-1.02; *P* = .004) when treating PM_2.5_ exposure as a continuous variable.

**Conclusions and Relevance:**

In this study, elevated zip code–level ambient PM_2.5_ exposure was associated with an increased hazard of death or graft failure in lung transplant recipients. Further study is needed to better understand this association, which may help guide risk modification strategies at individual and population levels.

## Key Points

**Question:**

Is living in an area with higher exposure to ambient fine particulate matter (PM_2.5_) air pollution associated with worse outcomes in patients with lung transplants?

**Findings:**

In this cohort study that included 18 265 adult lung transplant recipients, residence in a zip code with a mean annual PM_2.5_ concentration higher than 12μg/m^3^ was associated with an 8% increase in the hazard of death or graft failure.

**Meaning:**

This study found that lung transplant recipients who lived in areas with increased exposure to PM_2.5_ air pollution were at a higher risk of worse survival after lung transplant.

## Teaser

This cohort study investigates the association of neighborhood fine particulate matter air pollution exposure with death or graft failure among lung transplant recipients.

## Introduction

Lung transplant is an effective treatment for a carefully selected subset of individuals with a range of end-stage lung diseases. It is designed to be a life-prolonging intervention for patients with irreversible terminal lung conditions, but life expectancy after transplant remains modest, with a median survival of 5.5 to 6.5 years.^1,2^ There is a dearth of research on the association of neighborhood-level and environmental factors with lung transplant outcomes.^3^ Air pollution is among the most important environmental factors associated with lung health. Ambient particulate matter (PM) pollution is estimated to be the sixth leading cause of global disease burden, with 4.14 million attributable deaths per year in 2019.^4^ Short-term exposure to elevated PM pollution has been associated with increases in hospitalization for cardiovascular and respiratory diseases, as well as cardiovascular, respiratory, and all-cause mortality.^5,6,7^ Elevated long-term PM air pollution exposure has been associated with population-level increases in deaths from natural causes, all-cause mortality, coronary heart disease hospitalizations and deaths, asthma incidence, and stroke risk.^8,9,10,11,12^

Fine PM (PM_2.5_) pollution consists of fine, inhalable particles with diameters 2.5 μm or smaller and can include particles from combustion reactions, organic compounds, metals, and other materials. Larger, coarse particles typically deposit in the tracheobronchial regions, while fine, inhalable particles deposit deeper in the lungs, reaching the areas where respiration occurs, and are more likely to translocate from the lungs to the blood.^13,14,15^ Several studies have demonstrated that PM_2.5_ levels were associated with greater increases in poor health outcomes compared with PM_10_ levels (PM air pollution with a diameter <10 μm).^16,17,18^ Increased PM_2.5_ exposure has also been associated with decreased lung function in adults and worse lung development in children.^19,20^ A 2014 meta-analysis^21^ demonstrated that a 10 μg/m^3^ increase in PM_2.5_ exposure was associated with a 1.04% increase in the risk of death and 1.51% increase in risk of respiratory death among hospitalized patients. PM_2.5_ exposure has also been associated with worse health outcomes during the COVID-19 pandemic. Among patients in a New York City cohort, elevated annual PM_2.5_ exposure levels at the residential address were associated with an 11% increase in risk of death and 13% increase in risk of intensive care unit admission.^22^

Very few studies have investigated the specific associations of air pollution with health in lung transplant recipients. The density of roadways near a patient’s residence or the proximity of the residence to roadways have been associated with the development of chronic lung allograft dysfunction (CLAD) and bronchiolitis obliterans syndrome (BOS).^23,24^ One study looking at a cohort of 5707 lung transplant recipients across 12 centers in Europe demonstrated an increase in all-cause mortality and an increased incidence of CLAD in recipients who were exposed to higher levels of PM_10_.^25^ Another study assessing 520 recipients in France demonstrated that higher 1-year mean exposure to PM_2.5_ and PM_10 _particulate matter air pollution was associated with worse lung function as defined by a decreased forced vital capacity.^26^ This association was largely attenuated by the use of macrolide antibiotics for treatment of and prophylaxis against BOS.^27,28,29^ To our knowledge, there have been no large cohort-based studies investigating particulate matter air pollution and its association with lung transplant outcomes in the US.

## Methods

### Data Sources

We retrospectively analyzed data from the United Network for Organ Sharing (UNOS) Standard Transplant Analysis and Research file, which is used by every transplant center in the US to register and track waitlist candidates and transplant recipients. Data are available on patient- and limited donor–level factors for every candidate who has been placed on a solid organ transplant wait list since the inception of UNOS in 1987; data included the patient’s 5-digit zip code of residence at the time of transplant, based on Organ Procurement and Transplantation Network data. We restricted our analysis to recipients transplanted between May 2005, when the Lung Allocation Score (LAS) for wait list prioritization was introduced,^30,31^ and December 2016, constrained by the availability of air pollution data. A total of 18 265 recipients are included in the analysis, with a last follow-up date of September 10, 2020 (see eFigure 1 in Supplement 1 for patient flowchart). Primary analyses were completed between September 2022 and May 2023. Study protocols were reviewed by the institutional review board of the University of California, Los Angeles; an exemption was granted and informed consent was waived because the study was not considered human participant research.

### Measures

**Figure 1.**
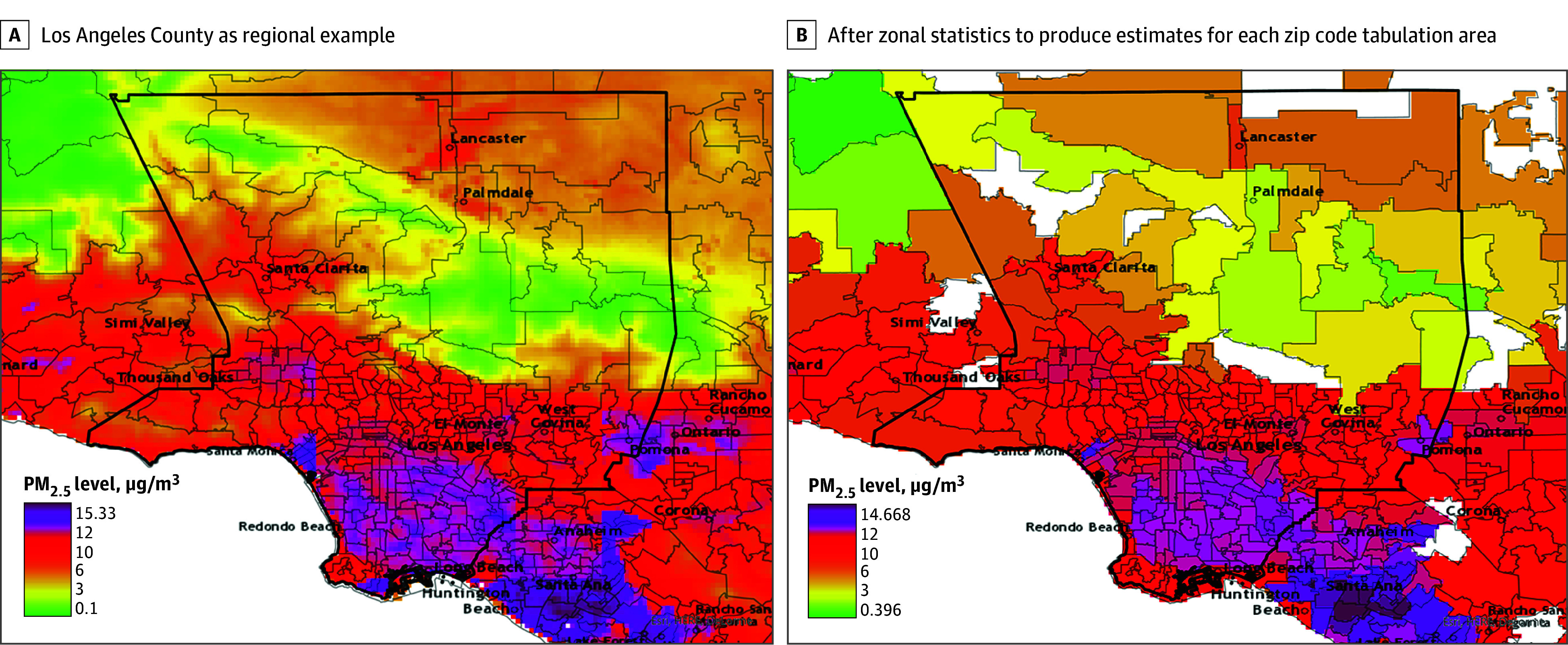
Mean Annual Concentration of Fine Particulate Matter (PM_2.5_) Pollution The mean annual concentration of particulate matter with an aerodynamic diameter of 2.5 μm or less is shown for Los Angeles County as a regional example (A) and after zonal statistics to produce estimates for each zip code tabulation area (B).

We constructed 5-digit zip code–level annual PM_2.5_ exposure estimates for each transplant recipient. Meng et al^32^ previously published yearly estimates of long-term PM_2.5_ concentration across North America. Their model combined data from chemical transport modeling, satellite-based measures, and ground-based measures to produce estimates of PM_2.5_ concentration at a resolution of 1 km × 1 km, with data available from 1981 to 2016. Using ArcGIS Pro version 3.1.0 (Esri), we overlaid each map of yearly estimated PM_2.5_ concentration with a shape file of zip code tabulation areas for the US. Using zonal statistics, we calculated the median PM_2.5_ estimate within the geographic boundary of each zip code tabulation area for each year of analysis (see Figure 1 for a regional example). Patient baseline PM_2.5_ exposure was obtained from the zip code of residence and year of transplant.

The outcome of interest was a composite of time to death or lung allograft failure, which allows the treatment of retransplant as a failure event. Other covariates included recipient age, sex, race and ethnicity, body mass index (BMI; calculated as weight in kilograms divided by height in meters squared), underlying lung disease category, single vs bilateral transplant, need for life support prior to transplant, LAS, type of health insurance, and medical center of transplant.^33,34,35,36,37,38^ Race and ethnicity were identified by the clinician, typically a nurse transplant coordinator, entering patient data into the UNOS registry. Race categories in the database were American Indian or Alaska Native, Asian, Black, Native Hawaiian or Other Pacific Islander, White, and race not reported. Ethnicity categories were Hispanic or Latino or not Hispanic or Latino. For this study, race and ethnicity were combined into a single field and American Indian or Alaska Native, Asian, Native Hawaiian or Other Pacific Islander, and race not reported were combined as other race and ethnicity. Race and ethnicity were assessed because differences in lung transplant outcomes by race and ethnicity have been reported in the literature and there are differences in exposure to PM_2.5_ air pollution by race and ethnicity. The need for life support was defined as the use of mechanical ventilation or extracorporeal membrane oxygenation (ECMO) at the time of transplant. LAS scores were grouped into categories of less than 35, 35 to 45, 45 to 55, and greater than 55. See eFigure 2 in Supplement 1 for our conceptual model.

### Statistical Analysis

Descriptive statistics were calculated at the patient level comparing the estimated mean annual zip code PM_2.5_ exposure greater than or equal to vs less than the Environmental Protection Agency (EPA) standard of 12 μg/m^3^. Continuous variables were compared between groups using Welch *t* test, while categorical variables were compared using χ^2^ tests. Time-to-event analyses were conducted for the composite end point of death or graft failure. Analysis was censored at 10 years of follow-up time. Kaplan-Meier survival curves were produced for high and low PM_2.5_ exposure levels, and a log-rank test was performed to determine equality of survival functions for comparison groups. Hazard ratios (HRs) for high PM_2.5_ were estimated using Cox proportional hazards models for unadjusted estimates and after adjustment for covariates. A Cox proportional hazards model including cluster-specific random effects at the level of the transplant center was performed using a gamma shared frailty model.^39^ The gamma shared frailty Cox proportional hazard model is a random-effects model that takes into consideration the observed heterogeneity from individual-level covariates, as well as the unobserved heterogeneity that is shared among observations belonging to a cluster, in this case the transplant center. Violations of the proportional hazards assumption were assessed visually by plotting Schoenfeld residuals against time and plotting the log-log survival time vs log time. All reported *P* values were 2-sided, and all tests were performed at a 2-sided 5% significance level. Statistical analyses were conducted using STATA/MP version 16.1 (StataCorp).

Because we were solely estimating PM_2.5_ levels at the year of transplant, we performed sensitivity analyses with censoring at 1 and 3 years to ensure that the association was consistent between medium- and long-term outcomes. We hypothesized that the association between PM_2.5_ and the hazard of death or graft failure would be linear. To explore this association, we conducted sensitivity analyses binning annual PM_2.5_ exposure into quartiles (Q1: ≤7.2 μg/m^3^; Q2: 7.2-8.6 μg/m^3^; Q3: 8.6-10.1 μg/m^3^; Q4: >10.1 μg/m^3^) and alternatively as a continuous variable in μg/m^3^. After the time of initial study conceptualization and analysis, the EPA has considered lowering the standard level, and in February 2024, the agency lowered the primary annual PM_2.5_ standard level to 9μg/m^3^. We conducted a post hoc sensitivity analysis using this new EPA standard as the cut point.

## Results

Among 18 265 lung transplant recipients (mean [SD] age, 55.3 [13.2] years; 7328 female [40.2%]; 1580 Black [8.7%], 1094 Hispanic or Latino [6.0%], and 15256 White [83.5%]), the zip code annual PM_2.5_ exposure was greater than or equal to the EPA standard of 12 μg/m^3^ for 1790 individuals (9.8%) and less than the standard for 16 475 patients (90.2%). Patient characteristics are provided in Table 1. Ages were similar between groups, with a mean (SD) age of 54.7 (12.7) years in the high and 55.4 (13.2) years in the low PM_2.5_ group. There were 752 females (42.0%) in the high and 6576 females (39.9%) in the low PM_2.5_ group. There was a statistically significant difference in the distribution of races and ethnicities between groups, with a higher proportion of Black and Hispanic or Latino transplant recipients and a lower proportion of White recipients in the high (247 Black [13.8%], 198 Hispanic or Latino [11.1%], and 1302 White [72.7%]) than the low (1333 Black [8.1%], 896 Hispanic or Latino [5.2%], and 13 954 White [84.7%]) PM_2.5_ group (*P* < .001). LAS score distribution was similar between groups. There were more recipients with private insurance (1107 individuals [61.8%] vs 8468 individuals [51.4%]) and fewer recipients with Medicare insurance (484 individuals [27.0%] vs 6382 individuals [38.7%]) in the high compared with the low PM_2.5_ group (*P* < .001). The zip code per-capita income was higher in the high PM_2.5_ group, with a mean (SD) of $34 485 ($16 876) compared with $33 535 ($13 406) in the low group (*P* = .02). There were fewer patients on ECMO or mechanical ventilation in the high PM_2.5_ group (110 individuals [6.1%] vs 1356 individuals [8.2%]; *P* = .002) and fewer patients who received bilateral transplants in the high PM_2.5_ group (1030 individuals [57.5%] vs 11 266 individuals [68.4%]; *P* < .001) compared with the low PM_2.5_ group.

**Table 1.** Baseline Patient Characteristics

Characteristic	Patients, No. (%) (N = 18 265)		*P* value^b^
	Zip code PM_2.5_ <EPA standard (n = 16 475 [90.2%])^a^	Zip code PM_2.5_ ≥EPA standard (n = 1790 [9.8%])^a^	
Age, mean (SD), y	55.4 (13.2)	54.7 (12.7)	.05
Sex			
Female	6576 (39.9)	752 (42.0)	.09
Male	9899 (60.1)	1038 (58.0)	
Race and ethnicity			
Black	13 954 (84.7)	1302 (72.7)	<.001
Hispanic or Latino	1333 (8.1)	247 (13.8)	
White	896 (5.2)	198 (11.1)	
Other^c^	292 (1.8)	43 (2.4)	
BMI, mean (SD)	25.1 (4.6)	25.0 (4.6)	.30
Diagnosis			
Obstructive pulmonary disease	4877 (29.6)	548 (30.6)	.02
Pulmonary vascular disease	503 (3.1)	52 (2.9)	
Cystic fibrosis	1932 (11.7)	165 (9.2)	
Restrictive lung disease	9163 (55.6)	1025 (57.3)	
LAS			
<35	4093 (24.8)	480 (26.8)	.15
>35-45	6127 (37.2)	667 (37.2)	
>45-55	2560 (15.5)	277 (15.5)	
>55	3695 (22.4)	366 (20.4)	
Insurance			
Private	8468 (51.4)	1107 (61.8)	<.001
Medicaid	1057 (6.4)	144 (8.0)	
Medicare	6382 (38.7)	484 (27.0)	
Other public	480 (2.9)	44 (2.5)	
Other	88 (0.5)	11 (0.6)	
Zip code per capita income, mean (SD), $^d^	33 535 (13 406)	34 485 (16 876)	.02
Mechanical ventilation or ECMO at time of match	1356 (8.2)	110 (6.1)	.002
Bilateral transplant	11 266 (68.4)	1030 (57.5)	<.001

Abbreviations: BMI, body mass index (calculated as weight in kilograms divided by height in meters squared); ECMO, extracorporeal membrane oxygenation; EPA, Environmental Protection Agency; PM_2.5_, fine particulate matter; LAS, Lung Allocation Score.

^a^ The EPA standard is 12 μg/m^3^.

^b^ *P* values are based on χ^2^ or Welch *t* test.

^c^ Other race and ethnicity includes American Indian or Alaska Native, Asian, Native Hawaiian or Other Pacific Islander, and race not reported.

^d^ The 2011 Income was reported using the 2019 Consumer Price Index adjustment.

**Figure 2.**
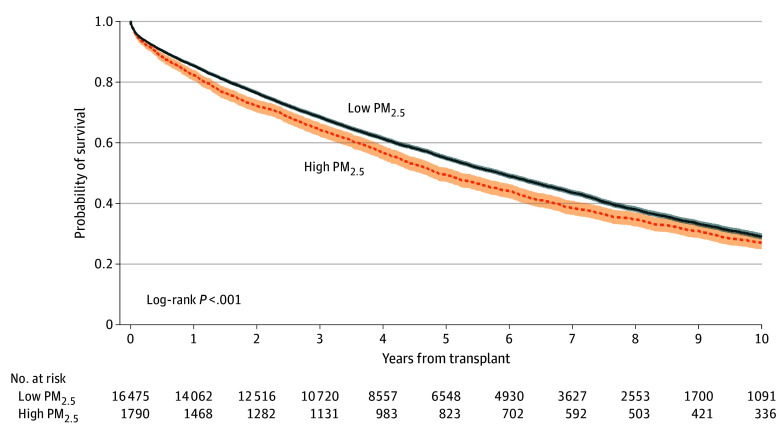
Kaplan-Meier Survival by Exposure Level Survival is shown for lung transplants within 10 years by fine particulate matter (PM_2.5_) exposure level. Shaded area indicate 95% CIs.

Kaplan-Meier survival curves for high and low PM_2.5_ groups are shown in Figure 2. A log-rank test with *P* < .001 demonstrated a statistically significant difference between the 2 survival functions. An unadjusted Cox proportional hazards model was fit and demonstrated that residence in a zip code with PM_2.5_ exposure greater than or equal to the EPA standard had an HR for death or graft failure of 1.11 (95% CI, 1.05-1.18; *P* < .001) compared with residence in a zip code with exposure less than the standard. The median graft survival was 4.87 years (95% CI, 4.57-5.23) years) for patients in high and 5.84 years (95% CI, 5.71-5.96) years) for patients in low PM_2.5_ areas.

Separate Cox proportional hazard models with gamma shared frailty around the transplant center were fit, adjusting for patient age, sex, race and ethnicity, BMI, insurance, underlying diagnosis category, use of mechanical ventilation or ECMO immediately prior to transplant, and single vs bilateral transplant (Table 2), with an HR for death or graft failure for high PM_2.5_ of 1.08 (95% CI, 1.01-1.15; *P* = .02). The data did not appear to violate the proportional hazards assumption by visual inspection plotting Schoenfeld residuals against time (eFigure 3 in Supplement 1) or plotting the log-log survival time vs log time (eFigure 4 in Supplement 1).

**Table 2.** Cox Proportional Hazards Models of Lung Transplant Death Or Graft Failure

Factor	Adjusted HR (95% CI)^a^^,^^b^	*P* value
PM_2.5_ level ≥EPA standard vs <standard	1.08 (1.01-1.15)	.02
Recipient age, per 10-y increase	1.03 (1.01-1.05)	.01
Female sex vs male sex	0.94 (0.91-0.98)	.005
Race and ethnicity		
Black	0.94 (0.87-1.01)	.10
Hispanic or Latino	0.87 (0.80-0.95)	.002
White	1 [Reference]	NA
Other^c^	0.89 (0.76-1.03)	.11
BMI, per 5-unit increase	0.99 (0.97-1.01)	.43
Mean per capita income, per $10 000 increase^d^	1.00 (0.98-1.01)	.62
Insurance		
Private	1 [Reference]	NA
Medicaid	1.26 (1.16-1.36)	<.001
Medicare	1.11 (1.07-1.16)	<.001
Other public	0.95 (0.83-1.08)	.41
Other	0.95 (0.71-1.27)	.74
Diagnosis		
Obstructive lung disease	1 [Reference]	NA
Pulmonary vascular disease	1.21 (1.07-1.36)	.002
Cystic fibrosis	1.04 (0.95-1.14)	.35
Restrictive lung disease	1.11 (1.06-1.16)	<.001
Life support^e^	1.42 (1.32-1.53)	<.001
Bilateral transplant	0.72 (0.69-0.76)	<.001

Abbreviations: BIC, bayesian information criterion; BMI, body mass index (calculated as weight in kilograms divided by height in meters squared); EPA, Environmental Protection Agency; HR, hazard ratio; NA, not applicable.

^a^ In the unadjusted Cox proportional hazards model, the HR for PM_2.5_ exposure greater than or equal to the EPA standard was 1.11 (95% CI, 1.05-1.18; P < .001). The bayesian information criterion was 191 851.2.

^b^ The adjusted model was a shared frailty Cox proportional hazards model, with a bayesian information criterion of 191 321.8. It was adjusted for age, sex, race and ethnicity, BMI, insurance, per capita income, diagnosis, mechanical ventilation or extracorporeal membrane oxygenation at time of match, and laterality.

^c^ Other race or ethnicity includes American Indian or Alaska Native, Asian, Native Hawaiian or Other Pacific Islander, and race not reported.

^d^ The 2011 income was reported using the 2019 Consumer Price Index adjustment.

^e^ Mechanical ventilation or extracorporeal membrane oxygenation.

**Figure 3.**
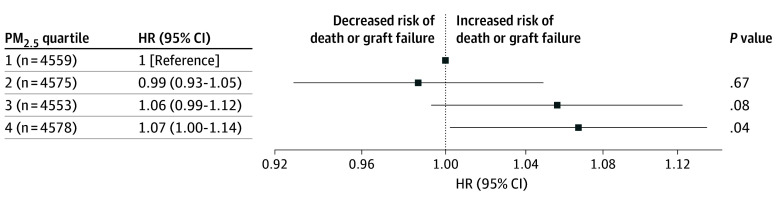
Risk of Death or Graft Failure by Exposure Quartile Forest plots are shown of the association estimate for the gamma shared frailty Cox proportional hazards complete model (covariates are age, sex, race and ethnicity, body mass index [calculated as weight in kilograms divided by height in meters squared], insurance, zip code per capita income, diagnosis, life support as use of mechanical ventilation or extracorporeal membrane oxygenation, and laterality) by fine particulate matter (PM_2.5_) exposure level. HR indicates hazard ratio.

The HR for the association of increased PM_2.5_ levels with mortality and graft failure increased when censoring was lowered to 3-year (HR, 1.17; 95% CI, 1.07-1.28; *P* < .001) and 1-year (HR, 1.27; 95% CI, 1.12-1.44; *P* < .001) follow-up (eTable 1 in Supplement 1). Refitting the main Cox proportional hazard model with gamma shared frailty respecifying annual PM_2.5_ exposure in quartiles demonstrated HRs for death or graft failure of 0.99 (95% CI, 0.93-1.05; *P* = .67) for quartile 2 and 1.06 (95% CI, 0.99-1.12; *P* = .08) for quartile 3, which were not statistically significant, and 1.07 (95% CI, 1.00-1.14; *P* = .04) for quartile 4 compared with quartile 1 (Figure 3; eTable 2 in Supplement 1). Kaplan-Meier survival curves for each quartile are displayed in eFigure 5 in Supplement 1, with the log-rank test demonstrating a statistically significant difference in survival functions. When treating annual zip code PM_2.5_ exposure as a continuous variable, each 1 μg/m^3^ increase in exposure was associated with an increase in the hazard of death or graft failure (adjusted HR, 1.01; 95% CI, 1.00-1.02; *P* = .004) (eTable 3 in Supplement 1). Changing the PM_2.5_ cut point to the new EPA standard of 9 μg/m^3^ yielded results consistent with our main analysis, with residence in a zip code with PM_2.5_ exposure greater than or equal to the EPA standard associated with an HR for death or graft failure of 1.07 (95% CI, 1.02-1.11; *P* = .003) compared with exposure less than the level after adjusting for covariates (eTable 4 and eFigure 6 in Supplement 1).

## Discussion

In this cohort study, we conducted a large registry analysis of lung transplant recipients to examine the association between zip code annual estimated PM_2.5_ exposure in the year of transplant and clinical outcomes. To our knowledge, this is the first large, US-based registry study assessing the association between ambient PM_2.5_ exposure and lung transplant outcomes. In our study, residence in an area with annual PM_2.5_ levels greater than or equal to the EPA standard was associated with an 8% increase in the hazard of death or graft failure and a 1% increase in the hazard of death or graft failure with each 1-μg/m^3^ increase in exposure. HRs for the association of air pollution exposure in the year of transplant with worse mortality or graft failure were higher when censoring at shorter follow-up. We expect that the true association of air pollution exposure with lung transplant outcomes may vary over time as air pollution exposure changes. We intended this analysis to determine whether the exposure in the year of transplant was enough for an association with poor long-term outcomes.

Elevated PM_2.5_ exposure is known to be associated with adverse health outcomes across multiple domains and cardiopulmonary conditions, but the mechanism of their associations in lung transplant is not well understood. Lung transplant has among the worst survival of all solid organ transplants, with the exception of intestine transplants.^40^ We hypothesize that this has to do with exposure to external elements, such as air pollution and microbes. More specifically, PM_2.5_ particles are small enough to reach the small airways and alveolar spaces where CLAD occurs (eg, BOS and restrictive allograft syndrome, respectively). We believe that lung transplant recipients represent a group that is highly susceptible to health outcomes associated with PM_2.5_, but this requires further research to prove mechanistically.^41^ PM_2.5_ exposure has been associated with short- and long-term adverse clinical outcomes in small lung transplant studies. Short-term increases in coarse PM exposure were associated with increased lymphocytic bronchiolitis on biopsies,^42^ which has in turn been associated with the development of CLAD.^43^

We theorize that the mechanism of ambient particulate air pollution exposure’s negative association with lung transplant is multifactorial, including direct injury to the lung via particle deposition and absorption, systemic inflammatory response, and immune dysregulation. Exposure to PM_2.5_ increases inflammatory cell infiltration and hyperemia of the lungs, inflammatory cell count in the bronchoalveolar lavage fluid, proinflammatory mediators, and oxidative stress damage in rodent models.^44,45^ Air pollution exposure may also alter immune homeostasis given that exposure of mice to PM_2.5_ is associated with an increase in Th2 cytokines.^46^ We and others have demonstrated that Th2 cytokine interleukin 13 (IL-13) is elevated and active in bronchoalveolar lavage fluid and biopsy samples from patients with BOS and that increased Th2 immune response is associated with CLAD development in lung transplant recipients.^47,48^ Exposure to PM_2.5_ has also been shown to impair the differentiation of Treg cells and promote the differentiation of Th17 cells, which can in turn produce IL-17 and recruit neutrophils in the lung.^49,50^ Th17 immune response has been associated with allograft rejection and CLAD in rodent and human studies.^51,52,53^ Additionally, elevated PM_2.5_ is associated with increased circulating endothelial and lung-specific microparticles, suggesting subclinical endothelial injury and increased endothelial cell apoptosis,^54^ which may predispose to dangerous allosensitization in the lung transplant recipient.

Our findings suggest that the association between PM_2.5_ exposure and lung allograft failure may be dose dependent given that quartile analysis demonstrated higher HRs for quartiles 3 (8.6-10.1 μg/m^3^ PM_2.5_) and 4 (>10.1 μg/m^3^ PM_2.5_). This raises the question of whether decreasing PM_2.5_ exposure further below the EPA standard of 12 μg/m^3^ may have additional benefit for lung transplant survival. Fortunately, after the initiation of this study, the EPA proposed and enacted a new primary standard of 9 μg/m^3^.^55^

### Strengths and Limitations

This study has several strengths and limitations. The study used panel data to test the association between elevated particulate matter air pollution exposure and graft failure and mortality after lung transplant. The use of registry data allowed for complete capture of the population of lung transplant recipients in the US with very little missingness in our variables of interest. A major limitation of the study is the potential for misclassification error. Patient residence is specified at only the 5-digit zip code level in the UNOS registry, and 5-digit zip codes and their spatial representation, the zip code tabulation area, are a less than ideal measure of neighborhood. However, they are easy for study participants to recall and are commonly used in registries and research studies.^56^ While more granular 9-digit zip codes exist, these are not as commonly remembered by participants and were not available in our dataset. It is reassuring that we have demonstrated an association despite the added noise of a less granular measurement of neighborhood. Additionally, the registry does not capture if patients relocate their residence during follow-up, so estimation of longitudinal exposure is not possible. It bears mentioning that our study follow-up period extended 4 years beyond our latest available PM_2.5_ estimates and that overall annual mean PM_2.5_ levels have decreased over time within our available air pollution data. Despite this weakness, establishing an association between air pollution exposure at baseline may be of great clinical utility as it allows practitioners to identify an at-risk population at the time of transplant. There may be unmeasured confounders and an array of zip code–based adverse social determinants that are related to PM_2.5_ levels that may contribute to poor health outcomes. Another limitation is that the UNOS registry does not capture granular information on the chronic treatment of patients, so we were also unable to adjust for the intensity of chronic immunosuppression or the presence of macrolide therapy. Prior studies have theorized that macrolide administration may attenuate the association of air pollution exposure with lung transplant outcomes.^25,26^ The lack of macrolide use information would bias our results toward the null, so the demonstration of an association despite this omission is encouraging. We theorize that controlling for differences at the center level partially captured some of the variation in treatment. Additionally, the registry does not capture other short- or intermediate-term negative outcomes after lung transplant, such as primary graft dysfunction, acute rejection, or CLAD, which may be associated with air pollution exposure.

## Conclusions

This cohort demonstrated that elevated PM_2.5_ exposure at the zip code of residence at the time of transplant was associated with increased hazard of death or graft failure in the first large, multicenter registry analysis of its kind in the US. Further research is needed to better understand the mechanism behind this association and determine whether it is modifiable at individual or population levels.

## Notes

Supplement 1. **eFigure 1.** Patient flowchart **eFigure 2.** Conceptual model of factors associated with lung transplant outcomes **eFigure 3.** Test of the proportional hazards assumption for PM_2.5_ **eFigure 4.** Test of the proportional hazards assumption for PM_2.5_ **eTable 1.** Gamma shared frailty Cox proportional hazards model with differential censoring **eTable 2.** Gamma shared frailty Cox proportional hazards model for PM_2.5_ quartiles analysis **eFigure 5.** Kaplan-Meier survival within 10 years by PM_2.5_ quartile **eTable 3.** Cox proportional hazards models for PM_2.5_ continuous analysis, unadjusted and adjusted models **eTable 4.** Cox proportional hazards models of lung transplant death or graft failure for zip code level PM_2.5_ exposure, threshold 9μg/m^3^, unadjusted and adjusted models **eFigure 6.** Kaplan-Meier survival for lung transplants within 10 years by PM_2.5_ exposure level with 9μg/m^3^ cutoff

Supplement 2.
Data Sharing Statement
