# Supplementary material for: Ambient Air Pollution Exposure and Outcomes in Patients Receiving Lung Transplant
Source: JAMA Netw Open. 2024 Oct 17;7(10):e2437148. doi: 10.1001/jamanetworkopen.2024.37148 (PMC11581506; doi:10.1001/jamanetworkopen.2024.37148)
Supplement: Supplement 1. — eFigure 1. Patient flowchart eFigure 2. Conceptual model of factors associated with lung transplant outcomes eFigure 3. Test of the proportional hazards assumption for PM2.5 eFigure 4. Test of the proportional hazards assumption for PM2.5 eTable 1. Gamma shared frailty Cox proportional hazards model with differential censoring eTable 2. Gamma shared frailty Cox proportional hazards model for PM2.5 quartiles analysis eFigure 5. Kaplan-Meier survival within 10 years by PM2.5 quartile eTable 3. Cox proportional hazards models for PM2.5 continuous analysis, unadjusted and adjusted models eTable 4. Cox proportional hazards models of lung transplant death or graft failure for zip code level PM2.5 exposure, threshold 9μg/m3, unadjusted and adjusted models eFigure 6. Kaplan-Meier survival for lung transplants within 10 years by PM2.5 exposure level with 9μg/m3 cutoff [file jamanetwopen-e2437148-s001.pdf]

Supplemental Online Content

Amubieya O, Weigt S, Shino MY, et al. Ambient air pollution exposure and outcomes in patients receiving lung transplant. *JAMA Netw Open*. 2024;7(10):e2437148. doi:10.1001/jamanetworkopen.2024.37148

eFigure 1. Patient flowchart

eFigure 2. Conceptual model of factors associated with lung transplant outcomes

eFigure 3. Test of the proportional hazards assumption for $PM_{2.5}$

eFigure 4. Test of the proportional hazards assumption for $PM_{2.5}$

eTable 1. Gamma shared frailty Cox proportional hazards model with differential censoring

eTable 2. Gamma shared frailty Cox proportional hazards model for $PM_{2.5}$ quartiles analysis

eFigure 5. Kaplan-Meier survival within 10 years by $PM_{2.5}$ quartile

eTable 3. Cox proportional hazards models for $PM_{2.5}$ continuous analysis, unadjusted and adjusted models

eTable 4. Cox proportional hazards models of lung transplant death or graft failure for zip code level $PM_{2.5}$ exposure, threshold 9μg/m^3, unadjusted and adjusted models

eFigure 6. Kaplan-Meier survival for lung transplants within 10 years by $PM_{2.5}$ exposure level with 9μg/m^3 cutoff

This supplemental material has been provided by the authors to give readers additional information about their work.

© 2024 Amubieya O et al. *JAMA Network Open*.

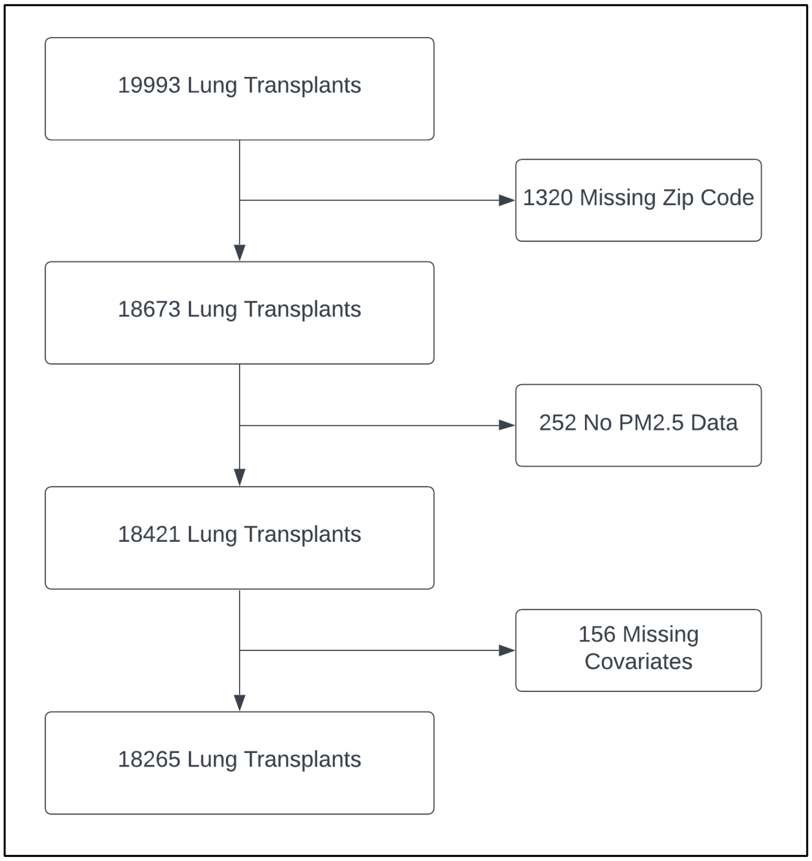

Figure S1. Patient flow chart

© 2024 Amubieya O et al. *JAMA Network Open*.

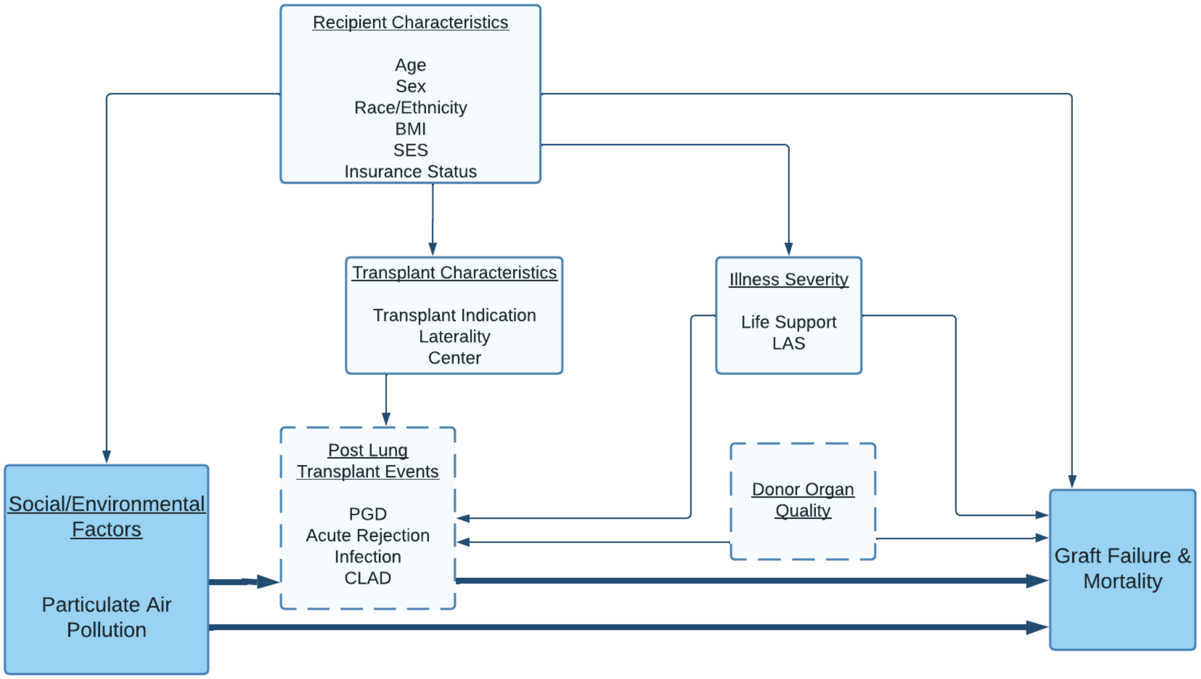

Figure S2. Conceptual Model of factors impacting lung transplant outcomes. Dashed boxes denote factors that are unmeasured in the dataset.

© 2024 Amubieya O et al. *JAMA Network Open*.

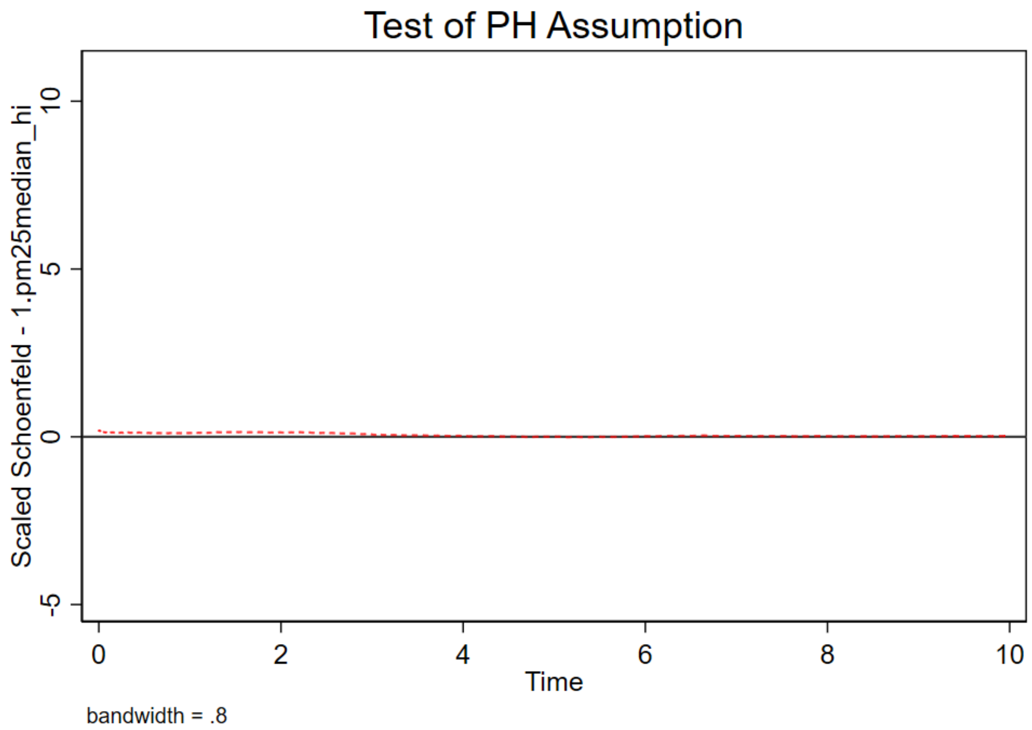

Figure S3: Test of the proportional hazards assumption for $PM_{2.5}$. Plot of Schoenfeld residuals versus time

© 2024 Amubieya O et al. *JAMA Network Open*.

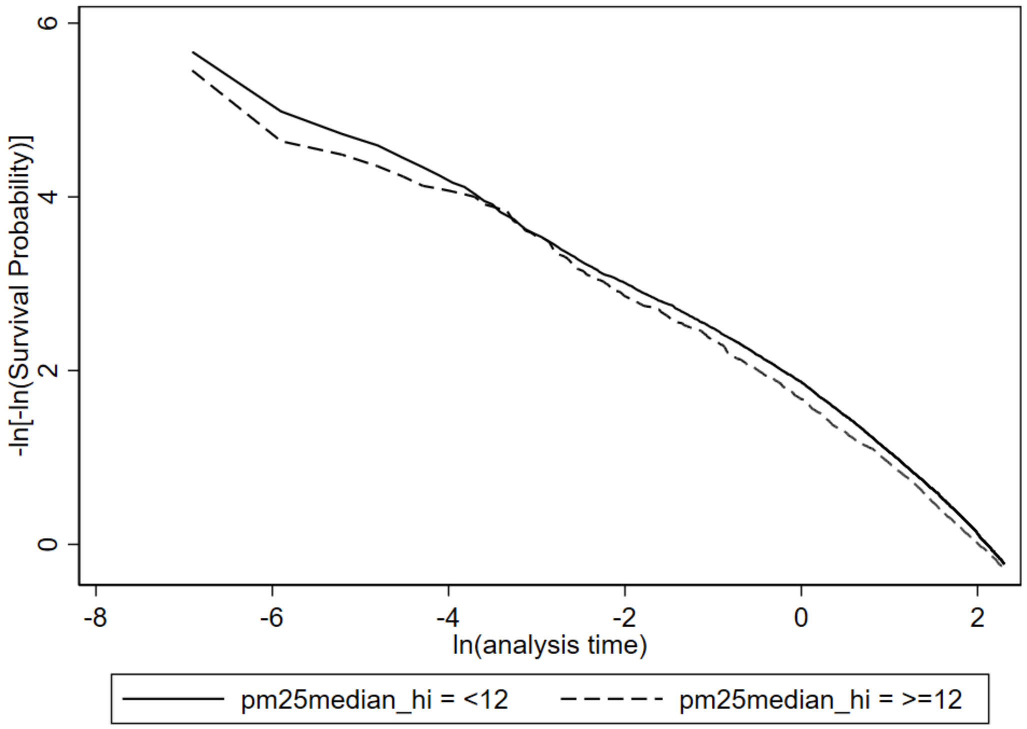

Figure S4: Test of the proportional hazards assumption for $PM_{2.5}$. Plot of log (log (survival probability)) versus log(time)

© 2024 Amubieya O et al. *JAMA Network Open*.

Table S1: Gamma shared frailty cox proportional hazards model with differential censoring

Differential Censoring	[a]1-Year Censoring		[a]3-Year Censoring	
Predictors	Hazard Ratio (95% CI)	p	Hazard Ratio (95% CI)	p
Model BIC	52499.9		111808.3	
$PM_{2.5}$ ≥EPA Standard	1.27 (1.12, 1.44)	<.001	1.17 (1.07, 1.28)	<.001
Recipient Age, *per 10 years*	1.08 (1.03, 1.13)	<.001	1.00 (0.97, 1.03)	0.98
Female Sex	0.86 (0.79, 0.93)	<.001	0.93 (0.88, 0.98)	0.005
Race				
White	ref		ref	
Black	0.96 (0.83, 1.11)	0.58	1.00 (0.91, 1.10)	0.94
Hispanic/Latino	0.89 (0.74, 1.05)	0.17	0.90 (0.80, 1.01)	0.07
[1]Other	1.01 (0.77, 1.33)	0.94	0.87 (0.71, 1.06)	0.17
BMI, *per 5 kg/m²*	0.98 (0.94, 1.03)	0.48	0.98 (0.95, 1.01)	0.15
[2]Per Capita Income, *per $10,000*	0.98 (0.96, 1.01)	0.31	1.00 (0.98, 1.02)	0.81
Insurance				
Private	ref		ref	
Medicaid	0.95 (0.80, 1.12)	0.53	1.15 (1.04, 1.28)	0.009
Medicare	1.02 (0.94, 1.11)	0.68	1.09 (1.03, 1.16)	0.002
Other Public	0.89 (0.69, 1.15)	0.39	0.91 (0.77, 1.09)	0.32
Other	0.75 (0.41, 1.36)	0.34	1.01 (0.70, 1.45)	0.97
Diagnosis				
Obstructive Lung Disease	ref		ref	
Pulmonary Vascular Disease	1.55 (1.24, 1.93)	<.001	1.34 (1.15, 1.57)	<.001
Cystic Fibrosis	0.99 (0.82, 1.20)	0.92	1.01 (0.90, 1.14)	0.87
Restrictive Lung Disease	1.29 (1.17, 1.42)	<.001	1.18 (1.11, 1.26)	<.001
[3]Life Support	2.10 (1.87, 2.37)	<.001	1.59 (1.46, 1.74)	<.001
Bilateral Transplant	0.88 (0.81, 0.97)	0.006	0.79 (0.74, 0.84)	<.001

[1]Other Race/Ethnicity includes American Indian or Alaska Native, Asian, Native Hawaiian or other Pacific Islander, and race not reported.
[2]2011 Income reported using 2019 Consumer Price Index (CPI) adjustment
[3]Mechanical Ventilation or extracorporeal membrane oxygenation (ECMO)
Adjusted for age, sex, race/ethnicity, body mass index (BMI), Insurance, per capita income, diagnosis, mechanical ventilation or ECMO at time of match, and laterality
[a]Shared frailty Cox proportional hazards model.
BIC - Bayesian information criterion; EPA - Environmental Protection Agency

© 2024 Amubieya O et al. *JAMA Network Open*.

Table S2: Gamma shared frailty cox proportional hazards model for $PM_{2.5}$ quartiles analysis

**$PM_{2.5}$ Quartiles** **Predictors**	**[a]10-Year Censoring** **Hazard Ratio (95% CI)**	**p**
Model BIC	191336.8	
$PM_{2.5}$ – 1st Quartile (≤ 7.2 µg/m^3)	ref	
$PM_{2.5}$ – 2nd Quartile (7.2-8.6 µg/m^3)	0.99 (0.93, 1.05)	0.67
$PM_{2.5}$ – 3rd Quartile (8.6-10.1 µg/m^3)	1.06 (0.99, 1.12)	0.08
$PM_{2.5}$ – 4th Quartile (> 10.1 µg/m^3)	1.07 (1.00, 1.14)	0.04
Recipient Age, *per 10 years*	1.03 (1.01, 1.05)	0.009
Female Sex	0.94 (0.91, 0.98)	0.006
Race		
White	ref	
Black	0.93 (0.87, 1.01)	0.07
Hispanic/Latino	0.87 (0.79, 0.95)	0.001
[1]Other	0.89 (0.76, 1.03)	0.11
BMI, *per 5 kg/m^2*	0.99 (0.97, 1.01)	0.42
[2]Per Capita Income, *per $10,000*	1.00 (0.98, 1.01)	0.55
Insurance		
Private	ref	
Medicaid	1.26 (1.16, 1.36)	<.001
Medicare	1.12 (1.07, 1.17)	<.001
Other Public	0.95 (0.84, 1.08)	0.45
Other	0.95 (0.71, 1.27)	0.75
Diagnosis		
Obstructive Lung Disease	ref	
Pulmonary Vascular Disease	1.21 (1.07, 1.37)	0.001
Cystic Fibrosis	1.05 (0.96, 1.15)	0.32
Restrictive Lung Disease	1.11 (1.06, 1.16)	<.001
[3]Life Support	1.42 (1.32, 1.53)	<.001
Bilateral Transplant	0.72 (0.69, 0.76)	<.001

[1]Other Race/Ethnicity includes American Indian or Alaska Native, Asian, Native Hawaiian or other Pacific Islander, and race not reported.
[2]2011 Income reported using 2019 Consumer Price Index (CPI) adjustment
[3]Mechanical Ventilation or extracorporeal membrane oxygenation (ECMO)
Adjusted for age, sex, race/ethnicity, BMI, Insurance, per capita income, diagnosis, mechanical ventilation or ECMO at time of match, and laterality
[a]Shared frailty Cox proportional hazards model.
BIC - Bayesian information criterion

© 2024 Amubieya O et al. *JAMA Network Open*.

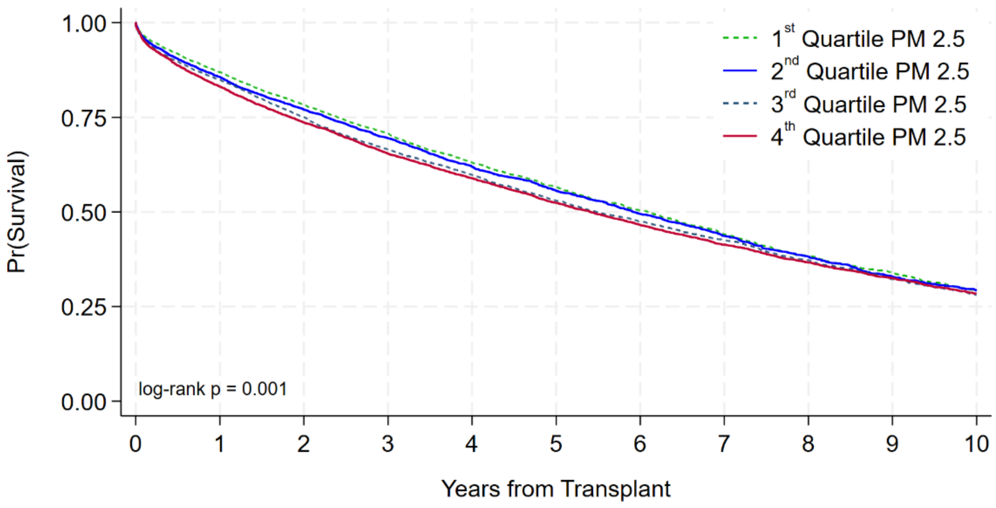

No. at risk

1st Quartile PM 2.5:	4,559	3,954	3,548	2,971	2,191	1,530	1,106	754	525	359	216
2nd Quartile PM 2.5	4,575	3,913	3,504	2,971	2,241	1,637	1,150	743	481	303	205
3rd Quartile PM 2.5	4,553	3,865	3,395	2,958	2,507	1,994	1,523	1,163	798	505	300
4th Quartile PM 2.5	4,578	3,798	3,351	2,951	2,601	2,210	1,853	1,559	1,252	954	706

Figure S5: Kaplan-Meier survival within 10 years by $PM_{2.5}$ quartile.

© 2024 Amubieya O et al. *JAMA Network Open*.

Table S3: Cox proportional hazards models for $PM_{2.5}$ continuous analysis, unadjusted and adjusted models

Continuous $PM_{2.5}$ Predictors	[a]Unadjusted Hazard Ratio (95% CI)	p	[b]Adjusted Hazard Ratio (95% CI)	p
Model BIC	191843.9		191318.9	
$PM_{2.5}$, *per 1 μg/m³*	1.02 (1.01, 1.03)	<.001	1.01 (1.00, 1.02)	0.004
Recipient Age, *per 10 years*			1.03 (1.01, 1.05)	0.008
Female Sex			0.94 (0.91, 0.98)	0.006
Race				
White			ref	
Black			0.93 (0.87, 1.01)	0.07
Hispanic/Latino			0.87 (0.79, 0.95)	0.001
[1]Other			0.88 (0.76, 1.03)	0.11
BMI, *per 5 kg/m²*			0.99 (0.97, 1.01)	0.42
[2]Per Capita Income, *per $10,000*			1.00 (0.98, 1.01)	0.55
Insurance				
Private			ref	
Medicaid			1.26 (1.16, 1.36)	<.001
Medicare			1.12 (1.07, 1.17)	<.001
Other Public			0.95 (0.83, 1.08)	0.43
Other			0.95 (0.71, 1.27)	0.73
Diagnosis				
Obstructive Lung Disease			ref	
Pulmonary Vascular Disease			1.21 (1.07, 1.37)	0.001
Cystic Fibrosis			1.05 (0.96, 1.15)	0.33
Restrictive Lung Disease			1.11 (1.06, 1.16)	<.001
[3]Life Support			1.42 (1.32, 1.53)	<.001
Bilateral Transplant			0.72 (0.69, 0.76)	<.001

[1]Other Race/Ethnicity includes American Indian or Alaska Native, Asian, Native Hawaiian or other Pacific Islander, and race not reported.
[2]2011 Income reported using 2019 Consumer Price Index (CPI) adjustment
[3]Mechanical Ventilation or extracorporeal membrane oxygenation (ECMO)
Adjusted for age, sex, race/ethnicity, body mass index (BMI), Insurance, per capita income, diagnosis, mechanical ventilation or ECMO at time of match, and laterality
[a]Cox proportional hazards model.
[b]Shared frailty Cox proportional hazards model.
BIC - Bayesian information criterion

© 2024 Amubieya O et al. *JAMA Network Open*.

Table S4: Cox proportional hazards models of Lung Transplant death or graft failure for ZIP code level $PM_{2.5}$ exposure Threshold 9μg/m^3, unadjusted and adjusted models

Predictors	[a]Unadjusted Hazard Ratio (95% CI)	p	[b]Adjusted Hazard Ratio (95% CI)	p
Model BIC	191851.3		191296.3	
$PM_{2.5}$ ≥EPA Standard	1.07 (1.03, 1.11)	<.001	1.07 (1.02, 1.11)	0.003
Recipient Age, *per 10 years*			1.04 (1.02, 1.07)	<.001
Female Sex			0.94 (0.90, 0.98)	0.002
Race				
White			ref	
Black			0.94 (0.88, 1.02)	0.12
Hispanic/Latino			0.88 (0.80, 0.96)	0.004
[c]Other			0.89 (0.76, 1.03)	0.11
BMI Category				
Underweight			1.27 (1.18, 1.37)	<.001
Normal			ref	
Overweight			1.01 (0.96, 1.05)	0.81
Obese			1.07 (1.01, 1.14)	0.02
[d]Per Capita Income, *per $10,000*			0.99 (0.98, 1.01)	0.45
Insurance				
Private			ref	
Medicaid			1.25 (1.15, 1.36)	<.001
Medicare			1.12 (1.07, 1.17)	<.001
Other Public			0.96 (0.84, 1.09)	0.49
Other			0.97 (0.73, 1.30)	0.84
Diagnosis				
Obstructive Lung Disease			ref	
Pulmonary Vascular Disease			1.22 (1.08, 1.38)	0.001
Cystic Fibrosis			1.02 (0.93, 1.12)	0.64
Restrictive Lung Disease			1.10 (1.05, 1.16)	<.001
[e]Life Support			1.42 (1.32, 1.52)	<.001
Bilateral Transplant			0.72 (0.69, 0.76)	<.001

Adjusted for age, sex, race/ethnicity, body mass index (BMI), Insurance, per capita income, diagnosis, mechanical ventilation or SD extracorporeal membrane oxygenation (ECMO) at time of match, and laterality
[a]Cox proportional hazards model.
[b]Shared frailty Cox proportional hazards model.
[c]Other Race/Ethnicity includes American Indian or Alaska Native, Asian, Native Hawaiian or other Pacific Islander, and race not reported.
[d]2011 Income reported using 2019 Consumer Price Index (CPI) adjustment
[e]Mechanical Ventilation or ECMO
BIC - Bayesian information criterion; EPA - Environmental Protection Agency

© 2024 Amubieya O et al. *JAMA Network Open*.

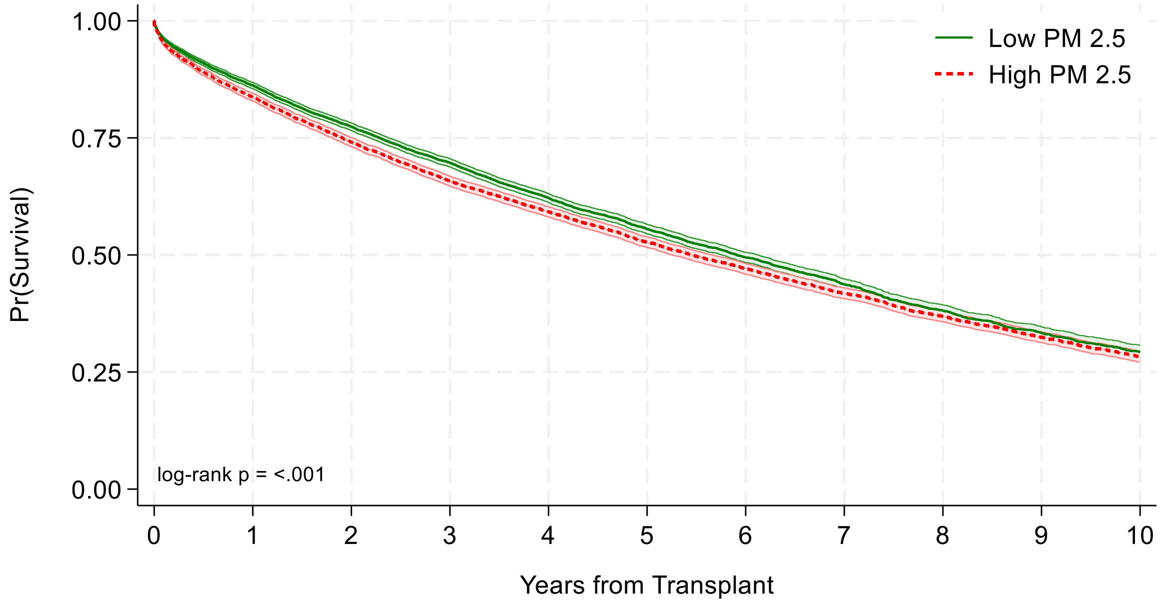

No. at risk

	0	1	2	3	4	5	6	7	8	9	10
Low PM 2.5:	10,475	9,014	8,054	6,800	5,140	3,698	2,634	1,778	1,187	769	485
High PM 2.5:	7,790	6,516	5,744	5,051	4,400	3,673	2,998	2,441	1,869	1,352	942

Figure S6: Kaplan-Meier survival for Lung Transplants within 10 years by $PM_{2.5}$ exposure level with 9μg/m^3 cutoff. Shaded area represents 95% confidence intervals

© 2024 Amubieya O et al. *JAMA Network Open*.
