# Supplementary material for: Ambient Air Pollution Exposure and Outcomes in Patients Receiving Lung Transplant
Source: JAMA Netw Open. 2024 Oct 17;7(10):e2437148. doi: 10.1001/jamanetworkopen.2024.37148 (PMC11581506; doi:10.1001/jamanetworkopen.2024.37148)
Supplement: Supplement 2. — Data Sharing Statement [file jamanetwopen-e2437148-s002.pdf]

Data Sharing Statement

Amubieya. Ambient Air Pollution Exposure and Outcomes in Patients Receiving Lung Transplant. *JAMA Netw Open*. Published online October 2, 2024. doi:10.1001/jamanetworkopen.2024.37148

Data
Data available: No

Additional Information
Explanation for why data not available: Per the data use agreement I used to access the United Network for Organ Sharing (UNOS) Registry, I am not allowed to share the data with other investigators. I would be happy to work with any interested investigator to work on their own data use agreement with UNOS to get access to the data to replicate this research.
